# Challenges in Commercializing Biomimetic Membranes

**DOI:** 10.3390/membranes5040685

**Published:** 2015-11-05

**Authors:** Mark Perry, Steen Ulrik Madsen, Tine Jørgensen, Sylvie Braekevelt, Karsten Lauritzen, Claus Hélix-Nielsen

**Affiliations:** 1 Aquaporin Asia, 1 Clean Tech Loop, CleanTech One #03-05, Singapore 63714, Singapore; E-Mail: mpe@aquaporin.asia; 2 Aquaporin A/S, Ole Maaløes Vej 3, Copenhagen N DK2200, Denmark; E-Mails: sum@aquaporin.dk (S.U.M.); tjo@aquaporin.dk (T.J.); sbr@aquaporin.dk (S.B.); 3 DSS, A Tetra Pak Company, Bergsøesvej 17, Silkeborg DK-8600, Denmark; E-Mail: Karsten.Lauritzen@tetrapak.com; 4 Faculty of Chemistry and Chemical Engineering, University of Maribor, Smetanova ulica 17, Maribor 2000, Slovenia; 5 Department of Environmental Engineering, Technical University of Denmark, Bygningstorvet 115, Lyngby 2800 Kgs., Denmark

**Keywords:** aquaporin membranes, biomimetics, commercialization, early stage technology

## Abstract

The discovery of selective water channel proteins—aquaporins—has prompted growing interest in using these proteins, as the building blocks for designing new types of membranes. However, as with any other new and potentially disruptive technology, barriers for successful market entry exist. One category includes customer-related barriers, which can be influenced to some extent. Another category includes market-technical-related barriers, which can be very difficult to overcome by an organization/company aiming at successfully introducing their innovation on the market—in particular if both the organization and the technology are at early stages. Often, one faces barriers from both these categories at the same time, which makes it necessary to gain insight of the particular market when introducing a new innovative product. In this review we present the basic concepts and discuss some of these barriers and challenges associated with introducing biomimetic aquaporin membranes. These include technical issues in membrane production and product testing. Then we discuss possible business models for introducing new technologies in general, followed by a presentation of beach-head market segments relevant for biomimetic aquaporin membranes.

## 1. Introduction

In today’s world, where water scarcity and water pollution pose imminent global threats to future economic development, countries, governments, companies, and individuals call for action. According to the United Nations World Water Development Report 2015 the world will experience a 40% deficit in water supply by 2030 [1]. Combined with the prediction that global demand for ‘blue’ water (*i.e.*, excluding water demand in rain-fed agriculture) will increase by 55% from 2000 to 2050, we face serious consequences if no actions are taken. By 2050 the global amount of blue water needed annually amounts to 5.500 km^3^. Although the predicted demand for irrigation water decreases slightly, it is more than surpassed in the three other main categories for water demand: electricity production, manufacturing and domestic use. In particular the global water demand for the manufacturing industry is expected to increase by 400% from 2000 to 2050 and the demand for electricity production is expected to more than double in the same period [1].

The increase in water demand is closely related to the fact that the world’s population is growing around 80 million people per year. It is predicted to reach 9.1 billion by 2050, with 2.4 billion people living in regions with highly heterogeneously-distributed water resources such as, sub-Saharan Africa. Combined with changing demographics with more and more people entering an affluent middle class, production of water-intensive foods and other nutritional products is increasing [2]. Thus, we face a triple-crunch from increased demands for water, energy, and food—often referred to as the water-energy-food nexus and viable solutions are strongly dependent on our ability to look beyond the water sector (and its segments) *per se* in order to develop specific water technologies.

**Figure 1:**
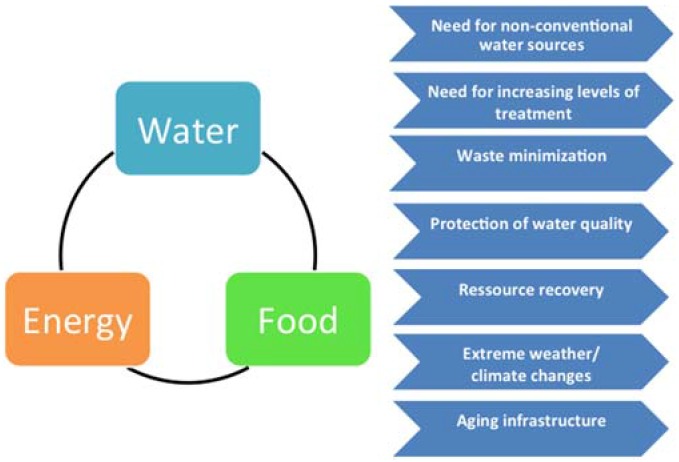
*Left:* The water-energy-food nexus, where provision of one element depends directly on the availability of the two others. *Right:* the challenges faced in the various water sectors. These can constitute key drivers for the development of innovative solutions and resilient technologies which can help in ensuring short- and long-term sustainability in water use.

One part of the crunch is obvious when we consider that although suitable technologies already exist that are providing solutions to almost all water-related challenges, they do require substantial amounts of energy to operate. For example, in desalination energy cost is the single largest factor in the cost of a seawater system (usually 20%–30% of the total cost of water [2]. Additionally, energy consumption is a large cost for municipal water distribution system utilities. Approximately 80% of municipal water processing and distribution costs are for electricity [3]. Thus, we find ourselves in a situation where, in order to solve the water challenges we have created for ourselves, we need energy to power water treatment systems. Couple this with the fact that accessible forms of energy are becoming increasingly scarcer—and, thus, more expensive—not to mention that an increasing amount of water related challenges originating from energy production itself and it is easy to see that we find ourselves in a vicious circle. The nexus challenges faced by the water industries can, in fact, be transformed into drivers for developing innovative and potentially disrupting technology solutions addressing the water-energy-food-nexus see Figure 1.

One driver is related to the deteriorated quality of water meant for production of blue water. In Europe for example, the Netherlands and Hungary are experiencing cross-border pollution where 46% of surface water reserves for Hungary are estimated to be affected [2]. This, in turn, leads to another driver arising from the need to use alternative and non-conventional water sources including desalinated seawater, non-potable/potable water recycling/reuse, and storm water capture. In addition, as the water treatment demand results in an increased quantity of waste, we must also begin to see waste, such as concentrated industrial wastewater, brine (e.g., from desalination), and sludge (e.g., from municipal treatment plants) more as a resource, with an associated research focus on resource recovery. Examples of this include harnessing thermal energy captured by heat-exchangers, as well as osmotic energy captured with membrane-based pressure-retarded osmosis. In the industrial sector, resource recovery could encompass non-product water reuse, for example, in cooling towers, boiler feed, and rinsing [4].

The above explains why increased productivity (*i.e.*, a higher ratio between treated water volume and the energy expended for the treatment process) is a major overall driver for developing and commercializing new water treatment technologies. Another general driver is quality. In order for increased productivity to be of value to the end user, the quality of the end product has to be up to par. Hence, new technologies delivering increased productivity—with adequate application specific quality of the end product—are of great demand in the water treatment market. It goes without saying that—in an unsubsidized market—new higher-productivity technologies are only of commercial interest if the payback times of the implemented solutions, *i.e.*, technological advancements, are shorter compared to existing solutions in the market.

## 2. Biomimetics as Inspiration for Membrane Technology

Drawing inspiration from nature’s own ways of transporting and purifying water is one particularly enticing strategy to boost productivity of water treatment technologies. Through billions of years of evolution, nature now provides an excellent palette of high-productivity membrane structures and materials capable of highly selective vectorial transport of a large number of molecular species [5]. In the approach often coined biomimicry or biomimetics such naturally-occurring materials are replicated, modified, and up-scaled to provide superior industrial products and solutions [6].

**Figure 2:**
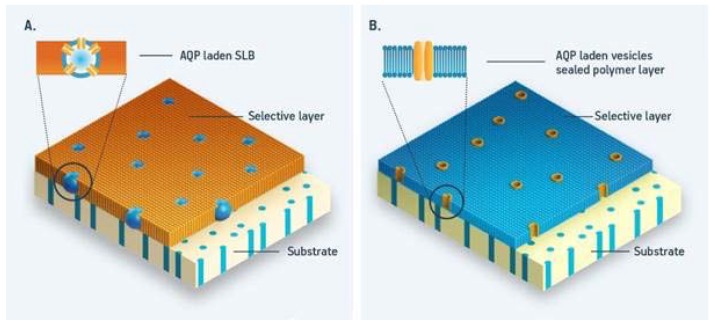
Schematic presentation of biomimetic aquaporin membrane designs (**A**): vesicles (blue) with embedded aquaporin protein (yellow) are immobilized in a polymer layer (orange) on a porous support substrate (beige); (**B**): supported membrane layer with aquaporin protein (yellow) is embedded in a flat layer (blue) deposited onto a porous support (beige). Adapted after [14].

Several biomimetic materials and processes are already being commercialized for industrial water treatment. One example of a biomimetic process is forward osmosis (FO), where specially designed semi-permeable membranes are used to facilitate diffusion of water in the same way water diffuses over biological membranes in nature, namely from low-solute concentration regions towards high-solute concentration regions. Through the last 30 years, the US-based company Hydration Technology Innovations (HTI) has pioneered the use of FO for various water treatment applications [7]. As a result of HTI’s efforts, the barriers for adopting (FO) technologies have steadily decreased to a point where a handful of companies, such as Oasys Water [8], Modern Water [9], Porifera [10], Trevi Systems [11], and Aquaporin A/S [12] are now commercializing FO systems for low-energy treatment of a wide range of challenging industrial wastewaters that were previously out of reach for traditional pressure driven membrane technologies. On the biomimetic membrane material side, Aquaporin A/S has developed the Aquaporin Inside™ Technology for both traditional reverse osmosis (RO) and cutting edge FO systems [13]. The membranes developed incorporate aquaporin proteins— nature’s own water channels resulting in higher membrane productivity and/or higher separation quality compared to conventional membrane technologies. Figure 2a schematically presents the Aquaporin Inside™ membrane design.

## 3. Up-scaling Challenges in Biomimetic Membrane Production

Many of the approaches for designing new membranes involve existing or new polymeric materials where most of these already are available in industrial quantities. In the case of biomimetic aquaporin membranes, the aquaporin protein is not yet a commercial commodity which constitutes the major challenge in up-scaling this technology. In general, production of membrane proteins is a non-trivial task for a number of reasons: first, protein expression in a host production organism often depends crucially on intracellular transport and modification pathways difficult to control; second, (over)-expression of membrane proteins may compromise cell membrane function, thereby having detrimental effects on the production host; third, protein purification often involve affinity-labelling and ultra-centrifugation, both of which may be difficult to scale up. Finally, the amphiphilic nature of cell membranes necessitates the use of carefully-selected detergents many of which are not currently available in bulk quantities. The need and associated cost for detergents alone can in fact be prohibitive for industrial scale (kilogram scale) of proteins, even if all other process steps are scalable and cost-effective. Recently, yeast-based systems have been investigated that may point the way to overcome some of these limitations [15,16]. Additionally, cell-free (CF) expression systems may be a way to diminish or eliminate toxic or inhibitory effects of the recombinant proteins on the host cell physiology and to avoid issues with having complicated transportation or translocation systems for the synthesized proteins [17]. Still, there are challenges associated with CF, such as the DNA degradation by endogenous nucleases in the cell extract.

In addition to the production of the aquaporin protein itself—a major challenge in upscaling biomimetic membranes originates from the fact that membranes capable of hosting membrane-spanning proteins must be able to hydrophobically match the proteins. This means that the host membrane must be in the order of a few nanometers thick, which again necessitates the use of integrated support materials. This inherent design requirement should be seen in the light of the operational demands for membrane applications *i.e.*, the demand for high permeability, high selectivity, sufficient mechanical stability to withstand the required pressure for target applications, chemical stability ensuring sufficient life time and stability towards cleaning procedures and, finally, ease of up-scaling.

So far two major design strategies have been employed in the design of biomimetic membranes; one based on integrating aquaporin containing vesicles in a suitable matrix, see Figure 2a, and one based on immobilized proteins in a planar membrane, see Figure 2b. The first design generally provides a mechanically robust membrane. The use of a dense polymer matrix in which the vesicles are embedded can further minimize defects, which in turn again enables potential industrial-scale production and protect aquaporins from chemical and biological attack. Although this approach, to some extent, limits the overall membrane water permeability, membranes still have a performance above average compared to conventional membranes. In addition, the production method closely resembles existing membrane fabrication methods—thereby lowering the market entry barrier. In the second design the ultrathin selective layer facilitates the fast transport of water molecules. Although potentially scalable, the low stability is an inherent and major drawback of this design. The key advantages and disadvantages of the two basic designs are discussed in detail in [14].

In any discussion of introducing and up-scaling new membrane technology it is important to realize the difference between the ability to scale up the membranes, which is closely related to the cost of the goods used to produce the membranes and the need to scale up production of membranes, which is directly related to the cost tolerated for the specific application. It is evident that for a large volume application, such as RO seawater desalination the new membrane performance and cost must be comparable with existing RO-based technology.

For conventional based seawater RO (SWRO) desalination CAPEX (capital expenditure) estimates are in the range of $1300–$2500 USD/(m^3^/d) [18]. The variability in the CAPEX range reflects that no distinction is made between plants contracted on an Engineering, Purchase, and Construction (EPC) basis or on a Build, Own, Operate, Transfer (BOOT) basis. In addition, the numbers vary due to variability in terms of seawater quality and plant capacity, site topography, and location. Additionally, the OPEX (operational expenditure) can vary and numbers for SWRO has been reported to be as low as $0.5 USD/m^3^ [18]. About half of this is electrical energy and about 6% represents membranes. However, labor and chemicals represents 37% of the OPEX, which reflects the need to maintain (clean) the membranes in operation. Thus, the ability to provide low-OPEX (*i.e.*, low-cost robust membranes with low fouling propensity) is crucial for a successful SWRO market entry for any new membrane technology.

On the other hand, water for hemodialysis is two orders of magnitude more expensive (with a required membrane lifetime in the order of hours), illustrating the strong potential of this high-end applications as a market entry for new membrane technology—both in terms of size requirements (thousands of m^2^ in an RO desalination plant *versus* small m^2^ sized membranes) for portable dialysis devices) but also in terms of cost of the water produced using the new membranes [14]. The extreme case in this respect is water treatment and reuse in space applications. The American National Aeronautics and Space Administration (NASA) has indeed investigated the possibility of using biomimetic aquaporin membranes and the results are promising [19].

## 4. Challenges in Launching a New Membrane Technology

Launching a new membrane technology possesses all of the challenges mentioned in the previous section. Water is essential to life and something we all need. Severe scarcity of water in many parts of the world has increased the awareness that water is a resource that must be treated with care. Whatever water treatment technology we choose to utilize it must, without a doubt, fulfil all requirements. 

Introducing a new process technology poses risks to the purchasing organization. Thus, it is essential that the risk can be reduced or eliminated. As no five-year reference list exists for biomimetic aquaporin membranes—since the technology is new—extensive testing is the best way to minimize risks. The sooner in the development phase of a new membrane technology one starts testing, the better. At Aquaporin A/S, a long-term test (one year) of FO and RO membranes were started as soon as the first prototypes of the Aquaporin Inside™ membrane were available and since 2012 pre-commercial membranes have been available for sale in coupons for testing.

A comprehensive testing program is needed in order to establish credibility. Through the testing, the membranes, in principle, must be exposed to every imaginable operating condition. The membranes must not only be tested during a specific operating condition, say high temperature, the performance of the membrane must also be tested after being exposed to the specific condition. At Aquaporin A/S, the new Aquaporin Inside™ FO membranes have been tested under a range of different operating conditions. Some of the basic testing conditions tested include:

- pH resistance. Membranes exposed to pH 2 and pH 11 followed by a performance test.
- Temperature resistance. Membranes exposed to 65 °C for 20 h followed by a performance (water flux and salt rejection) test.
- Storage. Test of membrane performance as a function of shelf life.
- Test of compatibility of the FO membrane with different types of draw solutions.
- Cleaning. Test of various detergents and anti-scaling agents.

With respect to temperature and pH resistance aquaporin membranes have been tested in the early stages of membrane development and the results are summarized in Table 1 and Table 2, see also [20]. The results show that the membranes can tolerate pH values as low as 2 and as high as 11 and temperatures as high as 65 °C and as low as 10 °C. The water flux becomes reversibly reduced during high and low pH and temperature feed values. Table 1 shows that the membranes are pH sensitive and tolerant with membrane performance *J_w_ J_s_* and calcein rejection *R*_Calcein_ being reversible when the feed solution was returned to neutral pH after 1200 min exposure to high/low pH. Table 2 shows that the membranes are heat tolerant for periods of 900 min exposure within the temperature range investigated although with changes in performance. At 65 °C higher water fluxes are accompanied by higher reverse salt fluxes, whereas operation at 10 °C results in a lower water flux and increased retention compared to performance at room temperature.

**Table 1:** Effect of high and low pH feed values. Tests were made with 1 L Milli-Q water spiked with 5 µM calcein (MW 622.55, Sigma, Aldrich, MO, USA) as feed solution and 2 M NaCl as draw solution. Measurements of water flux, reverse salt flux (conductivity), and calcein rejection (fluorometry) were performed on a membrane coupon mounted in a CF042 Sterlitech chamber (Sterlitech Corporation, Kent, WA, USA) with the active layer facing the feed solution and a counter crossflow rate of 50 mL min^−1^ corresponding to 0.85 cm min^−1^. Values are presented as mean +/− standard deviation over 1200 min with a sampling rate of 5 min^−1^ (*n* = 3).

Feed	n	*J_w_* (L m^−2^ h^−1^)	*J_s_* (gmh)	*J_s_/J_w_* (g L^−1^)	*R*_Calcein_ (%)
pH 7	14	12.60 ± 1.21	3.88 ± 0.83	0.31 ± 0.05	99.80 ± 0.22
pH 2.0	3	5.60 ± 0.79	-	-	-
Re-run at pH 7	3	12.22 ± 0.95	4.32 ± 0.26	0.35 ± 0.01	99.71 ± 0.19
pH11.0	3	7.44 ± 0.57	-	-	-
Re-run at pH 7	3	11.49 ± 2.42	4.17 ± 0.49	0.38 ± 0.12	99.55 ± 0.16

**Table 2:** Effects of high and low operational temperatures. Experiments were made as described for Table 1. Values are presented as mean +/− standard deviation over 900 min with a sampling rate of 5 min^−1^ (*n* = 3).

Temperature	*n*	*J_w_* (L m^−2^ h^−1^)	*J_s_* (gmh)	*J_s_/J_w_* (g L^−1^)	*R*_Calcein_ (%)
Reference 22°C	14	12.60 ± 1.21	3.88 ± 0.83	0.31 ± 0.05	99.80 ± 0.22
Heated to 65°C	3	22.09 ± 3.93	7.49 ± 3.4	0.33 ± 0.11	99.75 ± 0.29
Re-run at 22°C	1	11.55	4.08	0.35	99.81
Heated to 50°C	3	20.16 ± 6.20	3.67 ± 2.41	0.19 ± 0.11	99.92 ± 0.06
Re-run at 22°C	1	12.37	2.43	0.20	99.70
Cooled to 10°C	3	7.02 ± 0.16	2.43 ± 0.89	0.34 ± 0.12	99.95 ± 0.02
Re-run at 22°C	1	13.16	3.30	0.25	99.95

In order to determine storage/shelf times for membranes, samples fabricated according to the procedures described by Zhao *et al.*, have been subjected to drying at 50 °C for 1 min after fabrication and then stored in welded bags at 4 °C until the day of use.

**Figure 3:**
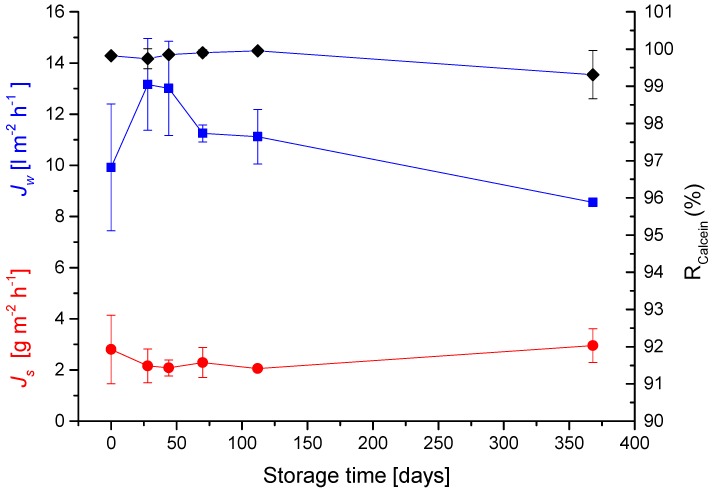
Effects of long term storage. Experiments were performed as described for Table 1. Sterlitech chamber with the active layer facing the feed solution. Values are presented as mean +/− standard deviation over 900 min (*n* = 3).

The results shown in Figure 3 demonstrate that biomimetic membranes can be stored for at least a year with no significance decrease in performance.

**Figure 4:**
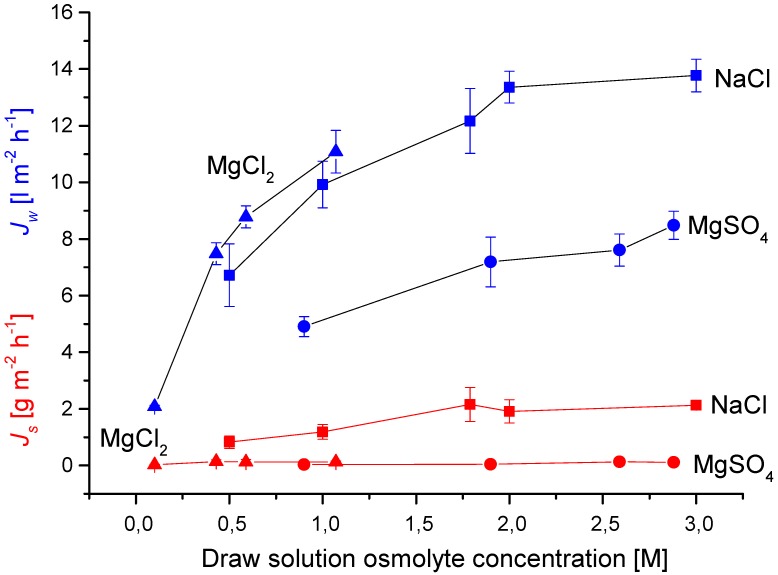
Effects of draw solution on water flux and reverse solute flux. Experiments were made as described for Table 1. Values are presented as mean +/− standard deviation over 900 min with a sampling rate of 5 min^−1^ (*n* = 3).

Another issue to consider in FO membrane operation is the choice of draw solution. One thing to consider is the stability of the membrane towards the draw solution per se. For draw solutions based on inorganic salts for example this may not be a serious issue *per se*. On the other hand the use of small monovalent ions may cause reverse salt flux exceeding acceptable limits. This could be the case for FO applications where the up-concentrated feed solution is the end-product. These applications include food and beverage (*i.e.*, up-concentration of fruit juice and wine most) and recovery of high-value products from dilute production streams. Additionally, the use of divalent ions may mitigate the issue of reverse salt flux which in turn may affect other membrane parameters such as fouling [21]. In order to examine this in more detail we performed FO experiments comparing mono- and divalent ions and the result are shown in Figure 4.

As Figure 4 shows the reverse salt flux values (measured as g m^2^ h^−1^) followed the order of NaCl > MgCl_2_ ≈ MgSO_4_. In terms of molar flux the order is NaCl (MW 58) > MgCl_2_ (MW 95) > MgSO_4_ (MW 120) reflecting that solute permeation through the membrane is affected by the properties of both cation and anion of the solute. The higher solute permeability of NaCl can be attributed to the smaller free energy of hydration energy (and ionic radius) of Na^+^ (−383 kJ mol^−1^) compared to Mg^2+^ (−1828 kJ mol^−1^). The difference in molar flux between MgCl_2_ and MgSO_4_ can likewise be attributed to the difference in free energy of hydration between Cl^−^ (−338 kJ mol^−1^) and SO_4_^2−^ (−1145 kJ mol^−1^).

With respect to chemical cleaning a wide variety of cleaning agents are used in membrane maintenance operations REF. Both organic and organic acids are effective cleaners for scale compounds and metal oxides. In general, cleaning efficiency is dependent on concentration, cleaning time, temperature, and hydrodynamic conditions. Thus, a comprehensive testing regime would entail a large set of testing conditions. Here, we will limit the examples to the use of citric acid and ethylene-diamine tetraacetic acid (EDTA).

The results shown in Table 3 demonstrate that the biomimetic membranes can be subjected to cleaning regimes at low and high pH without detrimental effects on the membrane performance.

**Table 3:** Effects of cleaning agents. Cleaning treatments were performed by circulating 0.3% CA (pH 2.2) and 0.8% EDTA (pH 9.0). Experimental conditions as given in the legend to Table 1. Each side of the membrane was treated with the treatment solution 50 ml/min for 15 min with a sampling rate of 1 min^−1^. Values are expressed as mean +/− standard deviation (*n* = 3).

Cleaning	*J_w_* (L m^−2^ h^−1^)	*J_s_* (g m^−2^ h^−1^)	*J_s_/J_w_* (g L^−1^)	*R*_Calcein_ (%)
Before CA treatment	10.33	2.26	0.44	99.94
After CA treatment	11.43	3.40	0.30	99.76
Before ETDA treatment	10.06	2.23	0.22	99.94
After EDTA treatment	10.99	3.51	0.32	99.00

Currently, the Aquaporin Inside™ flat sheet FO membrane performance data is shown in Table 4.

**Table 4:** Aquaporin Inside™ flat sheet FO membrane specifications as supplied by Aquaporin A/S.

Membrane Thickness:	110 µm (+/− 15 µm)
Water flux:	> 7 L/m^2^/h (H_2_O *vs.* 1 M NaCl; FO mode)
NaCl reverse flux:	< 2 g/m^2^/h (H_2_O *vs.* 1 M NaCl; FO mode)
Boron rejection:	>70%
Arsenic rejection:	>95%

Next step after laboratory testing is pilot testing. Pilot testing is necessary in order to prove the durability of the technology in an industrial setting. Pilot testing typically consists of small processing units that are placed in the location where it is planned to set-up a full scale system. By using the actual process water in your pilot system it will be possible to demonstrate that the system works in “the real world”. For some applications, other operating conditions are more suitable for tests; however, this must be evaluated for each case. Finally, there is the question of membrane lifetime. Biological membranes, *per se*, cannot be considered to be stable in the long term as continuous exchange of lipid molecules and proteins is a fundamental feature of such membranes. On the other hand, polymersomes with incorporated proteins embedded in a polyamide matrix (as shown in Figure 2a) can be seen as a very special version of a thin film composite membrane and it is plausible that stability of biomimetic membranes with this design is not significantly different from “classical” thin film composite membranes. Currently biomimetic membranes are only available in small scale—thus, it still remains to be seen how such membranes behave in long term operation. 

As a supplier of a new process technology it is also important to keep an open mind-set in the dialogue with your customer. One must listen carefully to any objections or concerns that might arise, and one must be prepared to change the specifications of your product to satisfy the customer. Most likely you can learn a lot from the customer comments. Through this relative intense dialogue with the customer you have the chance of establishing an added benefit; you have the chance to build integrity and credibility. At the end of the day, it requires a lot of trust to persuade an organization to bring in a new process technology. Engaging in a close dialogue with the customer is the only way to build this trust.

## 5. Business Models for the Introduction of a New Process Technology

Generally, introducing a new process technology in a business-to-business market presents a number of challenges. Most of the research that has been carried out regarding launching a new technology in general has focused on the business-to-customer market with less focus on the business-to-business market [22].

The decision making process when acquiring a new process technology is different from the decision-making process buying raw materials or consumables, as the impact is both more substantial and longer lasting. More stakeholders have to be taken into account and consequently the decision-making process when buying a new process technology is more complicated. When both the stakes are high and more stakeholders are involved in the process, it is likely that the whole decision-making process will have a high degree of risk aversion. Making a bold move might be perceived by individual stakeholders as a risk to their personal career prospects even though it makes perfect sense for the organization as a whole.

Risk can be moderated by being well prepared and by having the necessary funding available when launching a new production technology. In the pharmaceutical industry it has been found that firms that provide higher per-product levels of marketing and technology support obtain much greater financial rewards from their radical innovations than do other firms [23]. The complexity involved when launching a new production technology implies that it pays off to be very thorough in preparing market entry.

Choosing the right process technology will have significant economic effect for the purchasing organization. The consequences will be boosted by the fact that once a decision is made—to introduce a specific new technology—this decision cannot be changed for several years, because the consequences will only be experienced little by little over several years. The results of using a new process technology can only be discovered after a considerable amount of time as it takes time to run-in a new technology, adapt it to customer-specific circumstances, train operators, optimize the technology, *etc.* This means that by the time a possible mistake in the choice of technology is discovered, lots of resources have been wasted. Often the continuous optimization of a new process technology is the key to the success of the technology as the accumulated, continuous improvement is what creates superior performance in the long run. Needless to say, it is very hard to assess such future effects and, thus, the decision to introduce a new process technology is a decision that must cope with uncertainty.

As a consequence of the uncertainty, it is likely that the decision-making process of the key stakeholders becomes more conservative. In fact, it is likely that the risk aversion at the individual level in the organization becomes so prevalent that the decision-making process for the organization as a whole becomes seriously flawed. The risk of this is higher in some types of organization than in others. A large corporate organization where the decision making process is based on a certain degree of consensus is probably most at risk of not daring to take a calculated risk and go for a new process technology. An entrepreneurial-led organization is probably less likely to avoid risk-taking as risk-taking is often part of the self-belief of such organizations. Assessing the “risk mentality” of an organization can be helpful in order to evaluate whether an organization is likely to be well motivated to go for a new process technology.

## 6. Selection of Beach-Head Applications for Biomimetic Aquaporin Membrane Technology 

Most new technologies for water treatment applications will face high barriers to adoption. From an industry point of view, this is equivalent to high entry barriers for new technology providers entering the existing market space [24]; the reason being that proven technologies with long operational track records, are already being used to provide adequate and dependable treatment for the entire spectrum of water treatment applications. Hence, industrial end-users must be convinced on several parameters before they are willing to adopt new water treatment technologies:

1. Proven track record of OPEX and/or CAPEX savings compared to conventional technologies while taking into account all potential expenses of integrating the new technology with existing infrastructures.
2. Proven track record of long-term operational stability in similar industrial settings.
3. Technology suppliers must be well established both from a financial and production capacity point of view.

Providers of new technologies, who are not able to fulfil the points above, must select beach-head applications where industrial end-users face the highest pains using conventional technologies. In the case of the Aquaporin Inside™ Technology examples of such beach-head applications include:

- Aquaporin-based biomimetic FO membranes for dairy industries as a pre-treatment step to remove urea from process water streams. Here, conventional membrane technologies are unable to achieve sufficiently high urea rejections. For example RO membranes generally have urea rejections <40% [25].
- Aquaporin-based biomimetic FO membranes for NASA to recycle astronaut urine in space. Here, conventional technologies are either bulky (*i.e.*, expensive to transport into space) or unable to achieve sufficiently high urea rejections [19].
- Aquaporin-based biomimetic FO membranes for direct dewatering of challenging industrial wastewaters in biorefinery industries. Here, fermentation feed stocks and wastewater streams are currently handled independently. By using fermentation feed stocks as a draw solution to directly dewater wastewater streams through a FO process, significant resource and energy savings can be realized. Conventional RO membranes are not applicable in this case due to their propensity to foul.
- Aquaporin-based biomimetic RO membranes for suppliers of low-pressure household water purifiers. In this water treatment segment there is a constant consumer demand for more efficient and more compact purifier systems. Conventional RO membrane technologies have reached their performance limit and—as such—new technologies with higher productivity are needed to satisfy consumer demands.
- Aquaporin-based FO membrane assisted crystallization of Na_2_CO_3_, allowing its reuse, after CO_2_ capture from flue gases by an alkaline solution. By proper control of the super-saturation level high purity crystallization of Na_2_CO_3_·10H_2_O crystals can be achieved and aquaporin membranes may be a promising alternative to existing methods for Na_2_CO_3_ crystallization in a CO_2_ capture scenario [26]. The most developed technology today for CO_2_ capture is amine-based absorption. Despite high selectivity and loading capacity, a large amount of energy is needed for regeneration of the amine absorbents (about 3.5 GJ/ton CO_2_ removed) [27]. Thus, there is a need for more energy efficient solutions.

**Figure 5:**
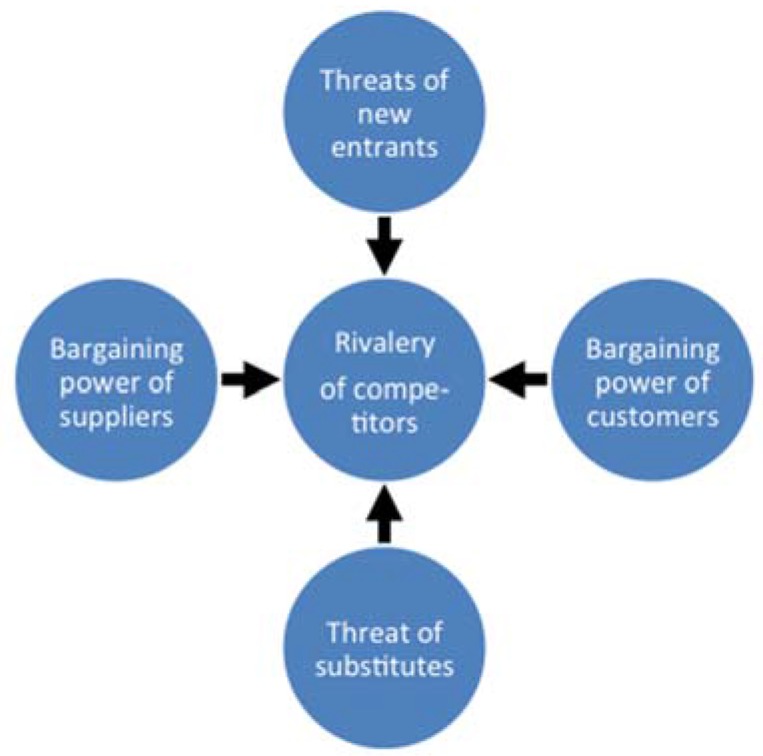
Porter’s five force analysis model for understanding where power lies in a business situation [24]. This model is used to analyze the strength of a current competitive position (e.g., between existing water technologies in the dairy industry), and the strength of a position one is considering moving into (*i.e.*, when introducing a new potentially disruptive technology such as biomimetic membranes).

To exemplify the key elements in Porter’s five force analysis [24], see Figure 5, we discuss these for the dairy beach-head application, since this application is an example where a small player with a new innovative technology interacts with large established players in a well-defined market. 

Bargaining power of suppliers: Important in the sense that a crucial component of the final product is a membrane protein, which currently is not commercially available. However several large-scale protein producers exist globally and this generally will tend to diminish the supply-risk associated with using the aquaporin protein in the membrane technology. All other membrane components used are commercially-available bulk commodities where the power of supplies must be regarded as very low.

Bargaining power of customers: In general, customer power is a strong force in dairy membrane applications, because it is an industry with big global players in the industry, and low/no need for service. For the dairy process water reclamation application the customer force is low, because the application is unique and covered by Intellectual Property Rights (IPR), therefore the customer can decide to proceed or not. Thus, if one can figure out the value of the application for a particular customer, one will have a good idea of her or his “walk away price”.

Threats of new entrants: This force is low in the dairy membrane industry, because there is a very conservative attitude towards processes and, thereby, supplier references. The marketplace is, in general, dominated by three large global competitors and a number of small local players.

Threat of substitutes: From a product point, this force in general is low in the dairy industry, because membrane filtration is a well-established technology, with high barriers of entry, because of high knowledge demand. In addition the industry is very technology-conservative. For the specific application (dairy process water treatment), no alternative technology has been recognized the last three years. Therefore, taking the IPR/first mover position and other factors into account this force is also believed to be low. From a technical point the application can be either based on RO or FO technology. RO membranes are a well-established technology—this may be an important threat as current RO membranes are low-cost commodities. FO is an emergent technology and the threat right now is less compared to RO. On the other hand the number of FO membrane producers is likely to increase in the near future which may increase the threat of substitute products based on FO technology.

Rivalry of competitors: The membrane industry is characterized by a few large players with little focus on research and development reflected in the percentage allocated to R and D of the overall revenue. A successful implementation (proof-of-market) of the biomimetic aquaporin technology can improve customer value (supporting higher prices) as it serves an unmet need (easy removal of small organic contaminants from process water). This may lead to an increase of the average profitability as new market segments emerge. Thus, an increased rivalry is to be expected in the future. 

Typically Porter’s five forces analysis model looks at both suppliers and customers as a kind of “enemies”. However, it will in many cases be an advantage to see customers and suppliers as partners with a common goal and thereby achieve some mutual common benefits. This is not possible if one constantly work against each other. The dairy beach-head application (process water reclamation) represents the entire value chain from component supplier to system contractor/end-user where one can maximize synergy by working and operating together while respecting stakeholder field and field-of-use definitions. At Aquaporin A/S, an open and collaborative philosophy forms the backbone of the commercial strategy. As a relatively small company launching a novel technology it is essential to establish Strategic Commercial Partnerships (SCP) with system contractors. A SCP is a long-term alliance, where both partners collaborate in order to optimize the use of a specific technology for a specific application or market. Aquaporin needs SCPs because end-users require complete systems, not just key components. In this sense, the stakeholders may in fact be considered as SCPs for the development of the biomimetic membrane technology. In this way, both parties commit to an investment in each other of time and money. From the point of view of the new innovative company, the risk in this investment is the uncertainty about how attractive the partner will be in the long run, *i.e.*, will the partner’s business grow or decline which will affect the demand for their technology. From the well-established partner’s point of view, the risk in this investment is the uncertainty about the performance of the new technology (*i.e.*, fouling propensity and membrane lifetime). The SCP must be seen as an investment from both parties’ point of view with a certain level of risk. The risk is accepted because both parties believe that the return on investment will cope.

Ideally, the five forces analysis sketched here should be viewed as a dynamic tool where one frequently revisits one’s position and the basis of competition. This, for example by identifying new/emerging segments where our technology is superior to existing technology and the competition thus is weaker. This will also help in minimize the risk of missing the opportunity to claim new strategic positions.

## 7. Perspectives and Outlook

The success of a new innovative product on the market can be hindered by one or more of the barriers and challenges discussed here. In order to overcome barriers for market introduction one can either focus on the barriers that can be influenced without much R and D effort or address barriers that have a relatively high chance to be dismantled successfully (e.g., by providing the necessary R&D). For biomimetic membranes the first type of barrier focus could arise from the fact that aquaporins are indeed the natural way of filtering water. This would then entail that added value comes from the technology being “green” by definition. For the second type of barrier focus, the superior performance in terms of flux and rejection of biomimetic membranes [13,28], may diminish market entry barriers in specific water market segments. By identifying segments where for example solute rejection is crucial, one may be able to identify customer needs currently unmet by conventional membrane technology. Finally, new barriers are likely to emerge in particular in environmental segments where policy driven changes may change the barrier landscape considerably. One example is the zero liquid discharge (ZLD) concept according to which essentially no liquid waste should leave the premises of an industrial unit [29]. The definition may be simple, but the implementation of ZLD can be complex. Thus, in a ZLD system it is vital that micro-contaminants, trace organics *etc.* in the pollutant streams do not pass through the treatment system thereby causing harm to human life, flora, and/or fauna in the natural habitat. Here, high rejection biomimetic membranes may in fact be a part of the solution—but no matter the segment—successful market entry depends on establishing and maintaining a good dialog with the customer—here not just regarded as a buying consumer but indeed as a strategic commercial partner.
